# Impact of Cooking Method on the Physicochemical Properties, Digestibility, and Sensory and Flavor Profiles of Chinese Chestnuts

**DOI:** 10.3390/foods14244331

**Published:** 2025-12-16

**Authors:** Lijun Song, Man Xu, Kai Zhang, Gang Guo, Lixiang Huai, Yue Zhao, Taohong Wang, Leiqing Pan, Ruiguo Cui, Li Zhang

**Affiliations:** 1 Chestnut Research Center, Hebei Normal University of Science and Technology, Qinhuangdao 066600, China; 2 College of Food Science and Technology, Hebei Normal University of Science and Technology, Qinhuangdao 066600, China; 3 Hebei Key Laboratory of Crop Stress Biology, Hebei Normal University of Science and Technology, Qinhuangdao 066600, China; 4 Hebei Food Inspection and Research Institute, Shijiazhuang 050227, China; 5 College of Food Science and Technology, Nanjing Agricultural University, No. 1 Weigang Road, Nanjing 210095, China

**Keywords:** nut, culinary treatment, microstructure, starch digestibility, volatile organic compounds

## Abstract

The impact of cooking method (stir frying, sugar stir-frying, baking, steaming, and boiling) on the physicochemical and sensory properties of Chinese chestnuts was evaluated. Dry heat treatment (stir frying, sugar stir-frying, and baking) increased hardness and chewiness because of water loss. Moist heat treatment (steaming and boiling) resulted in a softer texture and brighter color as a result of water absorption and starch gelatinization. Samples cooked with stir frying and boiling had a 50.82–54.17% reduction in resistant starch content. In contrast, the stir-frying, sugar stir-frying, and baking samples experienced a decrease of 37.16–47.18%. Concurrent changes in the glycemic index were observed. The polyphenol content and antioxidant activity were highest in the samples cooked using sugar stir-frying. A total of 34 volatile compounds were identified, but only 8 were key in the olfactory analysis (hexanal, (E)-2-hexenal, 3-methylbutanal, ethyl 3-methylbutyrate, ethyl acetate, 2-pentanone, 3-hydroxy-2-butanone, and 2-pentylfuran). At the same time, combined with sensory evaluation, sugar stir-frying can highlight the caramel and sweetness of chestnut; then baking can bring a strong aroma of nuts, and sugar stir-frying is a more popular method.

## 1. Introduction

The Chinese Chestnut (*Castanea mollissima* BL.), a woody food crop domesticated in China for more than 3000 years, is now widely cultivated across Asia and Europe [1]. Yanbao chestnut (*Castanea mollissima* BL. cv. ‘Yanbao’)—a late-maturing cultivar developed by our laboratory in the Yanshan range. The nuts are distinguished by a purplish-brown shell and creamy-yellow flesh rich in starch (27.10%), soluble sugars (9.42%), protein (10.50%), and fat (0.80%). In addition, it is also abundant in a variety of antioxidant substances, such as polyphenols (gallic acid, vanillic acid, etc.), flavonoids (chlorogenic acid, quercetin, etc.), procyanidins (catechin, epicatechin, etc.), and triterpenoic acid (chestnoside, maslinic acid, etc.), which exert protective effects against cardiovascular diseases, cancer, diabetes, etc. [2]. So it is particularly suitable for cooking. After cooking, it presents excellent sweet, fragrant, and glutinous qualities [3]. Yanbao is therefore well-suited for both fresh markets and processing applications [2].

A variety of culinary techniques are applied in chestnut processing, including stir-frying, sugar stir-frying, baking, steaming, and boiling. These methods remarkably influence the taste, flavor, bioactivity, and overall consumer acceptance of the final products [1]. To date, most studies have only examined the effects of cooking on the physicochemical properties of chestnuts, while investigations into starch digestion characteristics and flavor profiles remain limited. For instance, steaming and boiling induce starch gelatinization and protein structural changes, thereby enhancing the water- and oil-holding capabilities of chestnuts [4]. Baking, in contrast, causes pronounced alterations in color and structure [5] and disrupts starch crystallinity [4]. Stir-frying modifies the density and aspect ratio of the chestnut kernel [6], whereas sugar stir-frying, similar to baking, accelerates the Maillard reaction [7].

Cooking also modifies a wide range of bioactive components, including starches and polyphenols. Heat treatment, such as microwave heating, blanching, and steaming, has been reported to significantly increase both free and bound phenolic contents [8,9]. These increases in polyphenolic compounds are strongly correlated with enhanced antioxidant activity [2]. In addition, cooking methods can significantly affect starch digestion properties. For instance, steamed chestnuts exhibit a significantly higher GI than baked chestnuts [10]. Similar trends have been observed in legumes, where boiling and baking exert strong effects on in vitro starch digestibility [11].

Flavor represents another critical determinant of consumer preference. Chinese chestnuts are widely appreciated for their soft texture, sweet taste, and distinctive flavor [1]. However, studies focusing on the flavor characteristics of chestnuts, particularly across different cooking methods, remain scarce. Previous studies have shown that processing factors, such as packaging and thermal sterilization, can significantly alter chestnut flavor, with 28 out of 116 identified volatile compounds being strongly associated with specific treatments, as determined by PLS-DA [12]. In addition, decreases in moisture and protein content following baking and boiling influence aroma formation, with amino acids, such as arginine, threonine, and methionine, contributing to the characteristic chestnut flavor [13].

The objective of this study was to quantify the effect of five cooking methods (three dry heat and two moist heat) on the following properties of Chinese chestnuts: in vitro starch digestibility, antioxidant capacity, color and instrumental texture, and volatile organic compounds. The flavor profile was also evaluated by gas chromatography–ion mobility spectrometry (GC-IMS) and descriptive sensory analysis. The findings will provide theoretical support for technological advancements in the chestnut industry, thereby maximizing the nutritional and health benefits of chestnuts.

## 2. Materials and Methods

### 2.1. Materials and Chemical Reagents

Yanbao chestnuts were collected from Qinhuangdao city, Hebei province, China, on 25 September 2023. After collection, the samples were washed and drained of water, then vacuum packaged and stored at −4 °C for future use.

Ethanol, folinol, sodium carbonate, Folin–Ciocalteu reagent, DPPH (1,1-diphenyl-2-picryl-hydrazyl radical), ABTS (2,2′-Azino-bis (3-ethylbenzothiazoline-6-sulfonic acid)), and FRAP (Ferric ion reducing antioxidant power) were purchased from Solarbio Co., Ltd. (Beijing, China). The α-amylase, amyloglucosidase, and pepsin were purchased from Macklin Reagent. All other chemicals were analytical grade and purchased from Solarbio Co., Ltd. (Beijing, China).

### 2.2. Preparation of Chestnuts Using Different Cooking Methods

#### 2.2.1. Unified Shell Processing Operation

All chestnut samples were subjected to standardized shell treatment before cooking: the steaming and boiling treatment retained intact shells, while the stir-frying, sugar stir-frying, and baking treatments required shells to be manually opened with a knife, and the length of the opening was about 1/2 of the perimeter of the chestnut.

#### 2.2.2. Stir Frying of Chestnuts

Briefly, 500 g of treated chestnuts with a unified shell were mixed with 2500 g of quartz sand, and placed into a vertical frying machine (ZB-CHJ50LD, Guangdong Jiawang Baite Intelligent Technology Co., Ltd., Foshan, China), and stir-fried at 180 °C for 60 min. After cooking, the sample was removed and allowed to cool to room temperature (25 °C). Then, it was manually peeled, and the chestnut kernels were extracted for further analysis.

#### 2.2.3. Sugar Stir-Frying of Chestnuts

Similarly to Section 2.2.1, 500 g of treated chestnuts with a unified shell were mixed with 50 g maltose and 2500 g quartz sand and put into a vertical frying machine (ZB-CHJ50LD, China Guangdong Wang Bread Intelligent Technology Co., Ltd. Dongwan, China) and stir-fried at 180 °C for 60 min. The subsequent cooking procedures are consistent with those described in Section 2.2.1.

#### 2.2.4. Baking of Chestnuts

Briefly, 500 g of treated chestnuts with a unified shell were placed in an oven (Lecon, Domai Trading Co., Ltd., Shanghai, China) and baked at 130 °C for 40 min. The subsequent cooking procedures are consistent with those described in Section 2.2.1.

#### 2.2.5. Steaming of Chestnuts

Initially, water was brought to a boil. Subsequently, 500 g of treated chestnuts with a unified shell were placed on a steamer and cooked using a rice cooker (Triangle brand, Zhanjiang Household Appliance Industry Co., Ltd., Zhanjiang, China) for 30 min. The subsequent cooking procedures are consistent with those described in Section 2.2.1.

#### 2.2.6. Boiling of Chestnuts

Similarly to the procedure outlined in Section 2.2.4, water was initially brought to a boil. Subsequently, 500 g of treated chestnuts with a unified shell were placed into the boiling water and cooked in a rice cooker (Triangle brand, Zhanjiang Household Appliance Industry Co., Ltd., Zhanjiang, China) for 30 min. The subsequent cooking procedures are consistent with those described in Section 2.2.1.

### 2.3. Physicochemical Properties Determination

#### 2.3.1. Instrumental Texture Analysis

The texture characteristics (hardness, elasticity, chewiness, cohesiveness, and adhesiveness) of the samples were determined using a Texture Profile Analysis (TPA) test with a texture analyzer (CTX, Brookfield Corporation, TOR, New York, NY, USA). The processed chestnut shell was removed, and the chestnut kernel was cut into a block sample measuring 1 cm each in length, width, and height, which was then placed in the center of the analysis plate and a TA39 (cylindrical stainless-steel probe 2 mm in diameter) probe was used for testing. The test conditions are as follows: Set the distance between the sample and the probe at 30 mm, the compression ratio at 30%, the pre-test speed at 120 mm/s, the test speed at 60 mm/s, the post-test speed at 120 mm/s, the trigger force at 1 N, the cycle times to twice, with a time interval of 2 s, and each independent sample is subjected to three compression tests [14].

#### 2.3.2. Instrumental Color Analysis

The color was tested using a colorimeter (3nh, SC-10). The chestnuts were placed on a white sheet of paper, and then the instrument’s detection hole was aligned with the center of the chestnut for the test. The results were expressed as L*, a*, b*, and ∆E values. ∆E was calculated following Formula (1):

(1) $$∆\;E=\sqrt[{2}]{∆\;{L^{*}}^{2}+∆\;{a^{*}}^{2}+∆\;{b^{*}}^{2}}$$

where ∆E represents total color difference, and L, a, and b represent luminance, red–green degree, and yellow–blue degree, respectively.

#### 2.3.3. Moisture Content

The processed chestnut samples were ground, and then 2 g of the chestnut samples were weighed and dried in an oven (DHG-9245A, Shanghai Huitai Instrument Manufacturing Co., Ltd., Shanghai, China) at 105 °C until they reached a constant weight. The moisture content of the different chestnut groups was measured.

#### 2.3.4. Total Phenolic Content (TPC)

Firstly, the chestnut was crushed using a high-speed multifunctional grinder (KM-XA-1, Ningbo Kemai Instrument Co., Ltd., Ningbo, China), and then the chestnut powder was placed in an oven (DHG-9245A, Shanghai Huitai Instrument Manufacturing Co., Ltd., Shanghai, China) and dried at 35 °C until the weight became constant. The purpose is to eliminate the influence of moisture on the measurement results. After drying, the powder was passed through a 40-mesh sieve and stored for later use. Then, 1.0 g of chestnut powder was ultrasonically extracted using 10 mL of 50% ethanol under a power setting of 480 W for 20 min. The extraction process was repeated three times, and the supernatant was obtained by combining the supernatants from the three repetitions. After centrifugation, the supernatant was set aside. Using gallic acid as the standard substance, 1 mL of supernatant was placed into the test tube, and 5 mL of 10% Folin–Ciocalteu reagent was added. The test tube was then shaken well and placed at room temperature for 3 min. Then, 4 mL of 7.5% Na_2_CO_3_ solution was added, mixed well, and allowed to stand in the dark for 2 h. Subsequently, the absorbance was measured at 760 nm. The result is expressed as milligrams of gallic acid equivalent (mg GAE/kg) [15].

#### 2.3.5. Antioxidant Activity

The antioxidant activity was determined by referring to published methods [16]. Briefly, 1.0 g of chestnut powder was mixed with 10 mL of ethanol solution (50%, *v*/*v*), and extracted using an ultrasonic extractor (AK-080SD, Shenzhen Yujie Cleaning Equipment Co., Ltd., Shenzhen, China) at 35 °C for 20 min. The extraction was repeated three times. The supernatants were combined and concentrated in a vacuum for further determination.

The DPPH assay was conducted as follows: Firstly, the chestnut extract (500 µL) was diluted with methanol (5 mL). Then, 2.0 mL of the solution was mixed with 2.0 mL of DPPH solution, and reacted in the dark for 2 h. Finally, the absorbance was measured using a spectrophotometer (V-5800, Shanghai Yuanxi Instrument Co., Ltd., Shanghai, China) at 517 nm. The result was expressed as µmol Trolox/kg. The ABTS assay was conducted as follows: Firstly, the chestnut extract (500 µL) was diluted with methanol (5 mL). Then, 2.0 mL of the diluted solution was mixed with 2.0 mL of ABTS solution and reacted in the dark for 20 min. Finally, the absorbance was measured using a spectrophotometer (V-5800, Shanghai Yuanxi Instrument Co., Ltd., Shanghai, China) at 734 nm, and the result was expressed as µmol Trolox/kg. The FRAP assay was conducted according to the FRAP kit instructions. Briefly, the three solutions were mixed and preheated at 37 °C for 10 min. The chestnut extract (30 µL) was mixed with deionized water (90 µL) and the FRAP kit solution (900 µL). Then, the mixture was allowed to react at 25 °C for 10 min. Finally, the absorbance was measured using a spectrophotometer (V-5800, Shanghai Yuanxi Instrument Co., Ltd., Shanghai, China) at 593 nm. The result was expressed as µmol Trolox/kg.

### 2.4. In Vitro Digestibility of Starch

The in vitro digestibility of starch in chestnuts was detected using the Englyst and Infogest in vitro digestion models [17]. Using white bread as the reference, chestnut kernels (90 mg of starch equivalent) were suspended in pepsin solution (2000 U/mL) and incubated at 37 °C for 30 min to simulate gastric digestion. The intestinal digestion was then initiated by adding a sodium acetate buffer containing α-amylase (200 U/mL) and amyloglucosidase (100 U/mL). Samples were taken at specific time intervals within 240 min (0, 5, 10, 15, 20, 30, 60, 90, 120, 180, 240 min) at 37 °C, and then inactivated with ethanol. The absorbance was subsequently determined using a GOPOP kit at 510 nm. The following equations were used to calculate the contents of rapidly digestible starch (RDS), slowly digestible starch (SDS), resistant starch (RS), the digestion curve, and glycemic index (GI) value:

(2) $$\mathrm{RDS}(\%)=\frac{(G20-\mathrm{FG})\times 0.9}{\mathrm{TS}}\times 100$$

(3) $$\mathrm{SDS}(\%)=\frac{(G120-G20)\times 0.9}{\mathrm{TS}}\times 100$$

(4) RS(%)=100%−RDS−SDS

In these equations, G20 and G120 denote the glucose content (mg) following enzymatic hydrolysis for 20 min and 120 min, respectively. FG represents the content of free glucose (mg) in the starch before enzymatic hydrolysis. TS represents the total starch content (mg) in the sample.

(5) $$\mathrm{Digestibility}\;(\%)=A\times \frac{V_{1}\times C}{A_{0}}\times n\times V_{2}\times \frac{100\%}{m}\times \frac{162}{180}$$

(6) $$\mathrm{Starch}\;\mathrm{hydrolysis}\;\mathrm{index}\;(\mathrm{SHI})=\frac{\mathrm{IAUC}\;(\mathrm{Test}\;\mathrm{food})}{\mathrm{IAUC}\;(\mathrm{Reference}\;\mathrm{food})}\times 100$$

(7) Predicted GI=39.71+(0.549 ×SHI)

A represents the absorbance of the sample, A_0_ represents the absorbance of the standard product, V_1_ represents the volume of the sample, C represents the concentration of the standard product, n represents the dilution ratio, V_2_ represents the total volume of the reaction liquid, m represents the quality of starch, 162/180 represents the proportion of water loss of the glucose sample, SHI represents the hydrolysis index, and IAUC represents the area under the digestion curve.

### 2.5. Analysis of Microstructure by Scanning Electron Microscopy (SEM)

The morphological characteristics of raw chestnuts and chestnuts processed by different cooking methods were observed using a scanning electron microscope (SU8010, Hitachi Ltd., Tokyo, Japan). The chestnut powder was fixed onto the sample plate and then coated with gold to make the sample conductive. Digital images were acquired at ×2000 magnification with the built-in SEM-control software “Hitachi SEM Control & Capture (Version 2.1)”.

### 2.6. Flavor Profile Analysis

#### 2.6.1. E-Nose Analysis

A PEN-3 E-nose system (Airsense Analytics GmbH, Schwerin, Germany) equipped with ten sensors (Table S1) was used to measure the volatile components. The sliced chestnut (20 g) was placed into a headspace bottle (100 mL). The sample was then equilibrated at 30 °C for 30 min. Afterward, the system was purged for 120 s at a gas flow rate of 200 mL/min to ensure cleanliness. Subsequently, an automatic headspace sampler was used to introduce the aroma above the chestnuts into the electronic nose at a flow rate of 200 mL/min [18].

#### 2.6.2. Analysis of Volatile Organic Compounds by Gas Chromatography

Gas Chromatography–Ion Mobility Spectrometry (GC-IMS) analysis was conducted following a published method [19] using a FlavorSpec equipped with a CTC automatic headspace sampler ^®^ Flavor analyzer (G.A.S, BVB, Dortmund, Germany). Briefly, the chestnut sample (2.0 g) was placed into a headspace bottle (20 mL), after equilibration at 60 °C for 15 min, 500 μL gas was automatically injected for analysis.

The IMS conditions were as follows: nitrogen gas (purity ≥ 99.999%) was used as the carrier gas. The IMS temperature was 45 °C, with the gas flow initially maintained at 2 mL/min for 2 min. Subsequently, the flow rate was increased to 10 mL/min, and then ramped up to 100 mL/min over 20 min, and maintained it for 20 min. The length of the drift tube, linear voltage inside the tube, drift tube temperature, and drift gas flow rate were 53 mm, 500 V/cm, 45 °C, and 75 mL/min, respectively. After the instrument was turned on, 2-methyl-3-heptanone (1.0 µg/mL, internal standard) was continuously injected five times, and each sample was repeated three times. Volatile compounds were identified by matching the retention index (RI) and drift time (Dt) with the NIST/FlavorSpec database (version 2023.1) [20,21].

#### 2.6.3. Calculation of Relative Odor Activity Value (ROAV)

The relative odor activity value is used to assess the contribution of each compound to the overall aroma of the chestnut [22]. The formula of ROAV is as follows:

(8) $$\mathrm{ROAV}=\frac{\mathrm{Ci}}{\mathrm{Ti}}\times \frac{\mathrm{Tmax}}{\mathrm{Cmax}}\times 100$$

where Ci and Ti are the content and sensory threshold of volatile flavor components; Cmax and Tmax are the contribution content of overall volatile flavor components and the sensory threshold of the strongest overall volatile component.

### 2.7. Sensory Evaluation

The sensory evaluators were recruited from the School of Food Science and Technology, Hebei Normal University of Science & Technology. The evaluation team was composed of 10 preferred evaluators (6 females and 4 males, aged 18–25). These evaluators all possess the necessary techniques and practices in sensory description analysis, including attribute recognition and term development. Each evaluator sat in an independent booth, and the samples were randomly coded and presented in a one-time synchronous manner in tasteless paper cups. They were kept at 30 °C for 15 min and randomly presented to the evaluators. Then, the evaluators assessed the intensity of the odor through 5 descriptors (caramel, fatty, fruity, sweet, green, and overall score) and scored on a 10-point scale (10 being the highest, and 1, the lowest), and the average score was taken to draw a radar chart [13].

### 2.8. Statistical Analysis

All of the samples were tested three times in parallel, and the results were expressed as the mean ± SD. SPSS software 22.0 (IBM, Armonk, NY, USA) was used to analyze the data, and one-way ANOVA was used to test for significant differences (*p* < 0.05). The principal component analysis (PCA) for the electronic nose and GC-IMS data was conducted using Origin 2021 software (Origin Lab, Inc., Northampton, MA, USA). The orthogonal partial least squares discrimination analysis (OPLS-DA) and its Hotelling’s T^2^ test (*p* < 0.05) were both performed in SIMCA 14.1 (MKS Umetrics, Umea, Sweden). In addition, the software calculated the variable importance projection score (VIP), which evaluates the influence of each variable in the model based on its cumulative contribution to the explanatory X matrix (independent variable) and the predictive Y matrix (dependent variable). VIP > 1 is considered a significant contributing variable [23].

## 3. Results

### 3.1. Physicochemical Properties

#### 3.1.1. Texture and Color Properties

The textural attributes of chestnuts (hardness, adhesiveness, cohesiveness, springiness, gumminess, and chewiness) processed by different cooking methods are shown in Table 1. Compared with raw chestnuts, the hardness of chestnuts significantly decreased after heat treatment. The lowest hardness was exhibited by the boiled chestnut (33.17 N), followed by the steamed chestnut (38.39 N), the stir-fried chestnut (79.68 N), the sugar-stir-fried chestnut (84.75 N), and the baked chestnut (89.13 N). Stir frying, sugar stir-frying, and baking significantly reduced moisture, whereas steaming and boiling increased it, which is consistent with the results on chestnuts (*Castanea mollissima* BL.) of previous studies [14,21]. Possibly, the hardness observed after dry heat treatment (stir frying, sugar stir-frying, and baking) is higher than that after wet heat treatment (steaming and boiling), which can be attributed to the moisture loss during heating [14]. In contrast, steaming and boiling were not found to cause dehydration. Instead, the high temperatures facilitated the degradation of sugars, lipids, and proteins, thereby reducing hardness [12]. Moreover, heat-induced starch gelatinization disrupted crystalline structures, caused granule swelling, and prompted amylose leaching, ultimately forming a soft gel network that contributed to a decrease in hardness [24].

Adhesiveness was significantly lower in the sugar-stir-fried (0.02 N.mm) and baked (0.02 N.mm) chestnuts than in the steamed (0.04 N.mm) and boiled (0.04 N.mm) chestnuts. This difference may be explained by the increased porosity induced by heating [25]. Similarly, subsequent the SEM results also demonstrated that the sugar-stir-fried and baked chestnuts developed rough, folded surfaces with pores, a phenomenon also reported in stir-frying coix seeds [25].

The cohesiveness and springiness of stir-fried, sugar-stir-fried, and baked chestnuts are significantly higher than those of steamed and boiled chestnuts (*p* < 0.05). This is because, during dry heat treatments (such as stir-frying, sugar stir-frying, and baking), the water inside the chestnuts evaporated, and the starch partially gelatinized, thereby forming a tough gel network [6]. In contrast, wet heat treatments (including steaming and boiling) resulted in higher moisture content and more extensive starch gelatinization [14]. These findings are consistent with those observed in “Zao Feng” and “Da Ban Hong” chestnuts [21]. Meanwhile, previous research has shown that the chewiness of chestnuts is positively correlated with their hardness [14]. Our research also showed similar results, and the chewiness and hardness of chestnuts all decreased in the same order. Among the samples, the boiled chestnuts showed the lowest chewiness and hardness, which were 33.17 and 5.20, respectively, followed by the steamed chestnut, stir-fried chestnut, sugar-stir-fried chestnut, and baked chestnut, while the raw chestnuts showed the highest chewiness and hardness.

Color is another critical quality parameter that directly affects consumer acceptance. In this study, the L*, a*, and b* values were used to denote brightness, red–green intensity, and blue–yellow intensity, respectively, while ∆E indicated the overall color difference relative to raw chestnuts [21]. As shown in Table 1, the ∆E and L* values showed opposite trends. Raw chestnuts had the highest L* value (67.62), whereas processed samples ranged from 23.05 to 40.07, indicating a significant reduction in brightness after cooking (*p* < 0.05). “Zao Feng” and “Da Ban Hong” chestnuts also showed the same result [21]. During stir-frying, sugar stir-frying, and baking, the reduced moisture content and the increase in the relative concentration of protein accelerate the Maillard reaction, producing a darker appearance [26]. Conversely, steaming and boiling led to a decline in water-soluble protein content, which possibly reduced the degree of Maillard reaction and contributed to the relatively higher brightness of these samples [13]. The a* values of cooked chestnuts varied from 6.25 to 6.97, with no significant differences observed (*p* > 0.05). In contrast, raw chestnuts exhibited the highest b* value (19.20), whereas sugar-stir-fried chestnuts showed the lowest values (8.08). The b* values for stir-fried, baked, steamed, and boiled chestnuts were 8.59, 12.93, 13.52, and 10.95, respectively. These results indicate that the yellow–blue color component of chestnuts was significantly altered by thermal processing (*p <* 0.05). Such alterations can be attributed to thermal degradation at high temperatures, which promotes caramelization and browning reactions [27].

#### 3.1.2. TPC and Antioxidative Capacity

**Figure 1:**
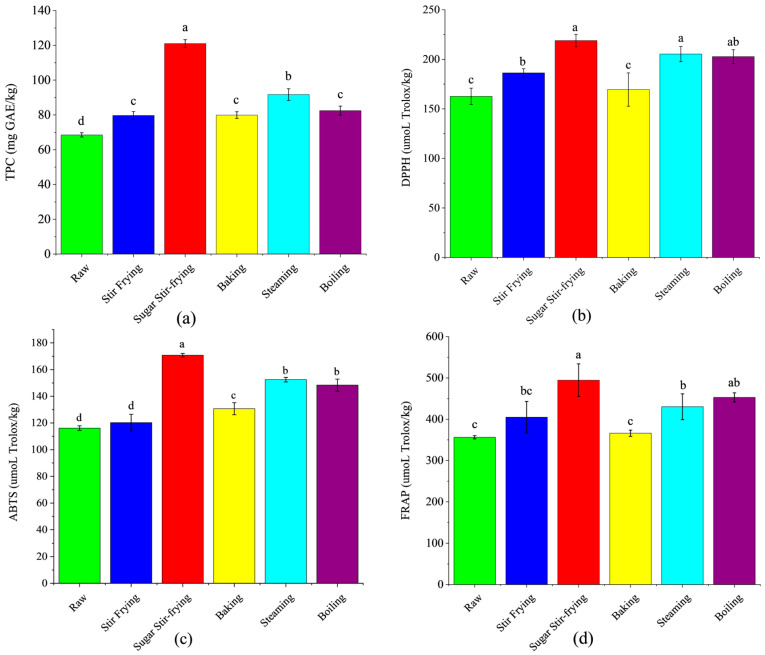
Impact of chestnut cooking method on (**a**) total polyphenol content (TPC), (**b**) 2,2-diphenyl-1-picrylhydrazyl (DPPH), (**c**) 2,2′-azino-bis(3-ethylbenzothiazoline-6-sulfonic acid (ABTS), and (**d**) Ferric Reducing Antioxidant Power (FRAP). Column with different letters are significantly different (*p* < 0.05).

Polyphenols are bioactive compounds with well-documented physiological functions, including antioxidant, anti-inflammatory, and hypoglycemic effects [7]. The effects of different cooking methods on the TPC and antioxidative capacities of chestnuts are shown in Figure 1. As shown in Figure 1a, the raw chestnut displayed the lowest TPC. Cooking significantly increased TPC (*p* < 0.05), ranging from 68.53 mg GAE/kg to 121.02 mg GAE/kg across treatments. Among the different methods, the sugar-stir-fried chestnuts showed the highest TPC (121.02 mg/kg), followed by steamed (91.71 mg GAE/kg), baked (79.89 mg GAE/kg), stir-fried (79.69 mg GAE/kg), and boiled chestnuts (82.40 mg GAE/kg). These results are consistent with previous studies on chestnuts [21,23,28], which, upon reporting the effects of heat treatment on phenols in chestnuts, noted that heat treatment promoted both the degradation and release of phenolic compounds, primarily through the thermal disruption of cell walls and the liberation of phenolic acid precursors [8]. In addition, heating may facilitate the diffusion of polyphenols from the shell layer to the kernel, further enhancing TPC [7]. Similar increases in TPC after heat treatment have also been observed in boiling wheat congee [29].

Antioxidant activities, defined as the capacity to scavenge free radicals and mitigate oxidative damage [29], were evaluated using DPPH, ABTS, and FRAP assays, as shown in Figure 1b–d. The cooked chestnuts demonstrated significantly stronger antioxidant activities than raw samples. The sugar-stir-fried chestnuts showed the strongest antioxidant activities, with DPPH, ABTS, and FRAP activities of 218.91, 170.74, and 494.51 μmol Trolox/kg, respectively. This enhancement can be attributed to the intensified Maillard reaction caused by the addition of maltose syrup during sugar stir-frying. The Maillard reaction produces numerous intermediate and end-products with antioxidant properties [30]. Specifically, hydrogen atom transfer and single-electron processes during the reaction can neutralize DPPH and ABTS^+^ radicals, while Maillard-derived compounds are also capable of chelating Fe^3+^ ions, thereby increasing FRAP values [31]. Consequently, heat treatments, particularly sugar stir-frying, markedly enhanced the antioxidant potential of chestnuts. These results are consistent with the observations in previous chestnut studies on the effect of heat treatment on the antioxidant activity of chestnuts [8,9]. Meanwhile, in roasted almonds, it was also found that the Maillard reaction products (such as melanoids) were positively correlated with free radical scavenging capacity [32].

### 3.2. In Vitro Starch Digestibility Analysis

**Figure 2:**
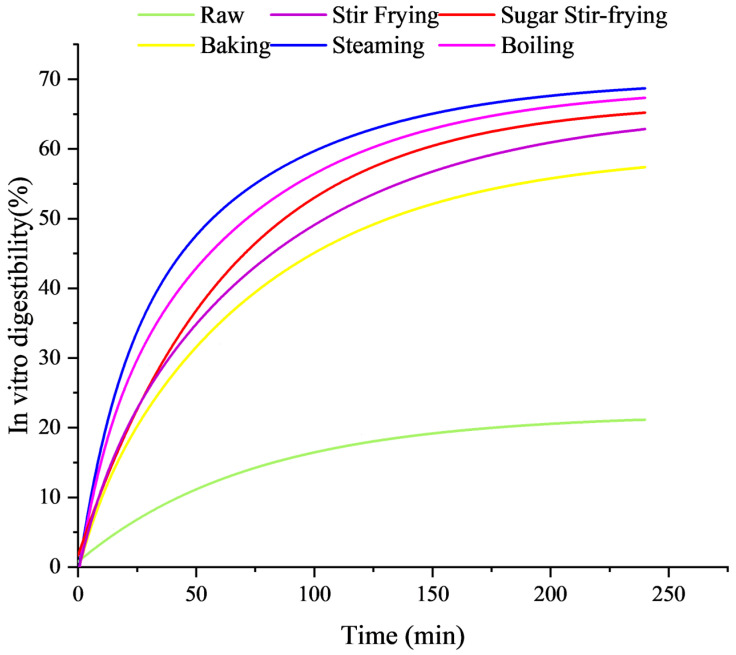
Impact of chestnut cooking method on in vitro starch digestibility.

Starch digestion may be influenced by water phase distribution, temperature, and intermolecular interactions [10]. As shown in Table 1 and Figure 2, different cooking methods have significant impacts on starch digestibility. Compared with raw chestnuts, the cooked chestnuts exhibited significantly higher contents of RDS and SDS (*p* < 0.05), and a significant decrease in RS (*p* < 0.05). Steamed chestnuts had the highest RDS content (29.95%) and the lowest RS content (37.58%). This observation is possibly due to high temperature and humidity facilitating starch–water interactions, accelerating gelatinization, disrupting crystalline structures, and promoting gelation, thereby enhancing digestibility [24]. In contrast, the content of RDS and SDS in stir-fried, sugar-stir-fried, and baked chestnuts increased slightly. This limited effect may result from the moisture loss and heat-induced formation of amylose–lipid complexes, which shield enzymatic cleavage sites and inhibit gelatinization [4]. During different cooking processes, the content changes in RS showed an opposite trend to the in vitro digestibility, which is consistent with the previous research results by Gao et al. on chestnuts [10].

The in vitro starch-digestion kinetics of different samples are shown in Figure 2. The steamed chestnuts exhibited the highest hydrolysis degree (about 68%), followed by boiled chestnuts (about 66%). Presumably, this slightly lower hydrolysis is due to soluble starch leaching into the cooking water [33]. The stir-fried, sugar-stir-fried, and baked chestnuts displayed similar hydrolysis patterns, while raw chestnuts displayed the slowest digestion because their starch granules and crystalline regions remained intact. These results align with the degree of particle fragmentation observed by SEM for raw chestnuts and chestnuts treated with different cooking methods and with the RDS/RS data (Table 1). Steaming and boiling yielded significantly higher digestion rates than baking, likely because the high temperature and water converted long-chain amylose into shorter fragments, which are more accessible to enzymes. This pattern is consistent with reports on soy starch [11] and oat starch [6], where steaming and boiling increased digestibility compared to baking. The superior digestibility and GI of steamed chestnuts may also result from water vapor permeation, which enhances enzymatic accessibility [34]. Stir-frying was found to induce partial gelatinization and amylose–amylopectin recombination, forming an amorphous structure that lowered enzymatic resistance [6]. During the sugar stir-frying process, maltose may undergo hydrolysis to form glucose. Therefore, compared with stir-fried chestnuts, sugar-stir-fried chestnuts have a higher digestibility and GI.

Steamed chestnuts exhibited the highest glycemic index (GI, 85.42), while raw chestnuts showed the lowest value (52.49). This elevated GI of steamed chestnuts can be attributed to complete starch gelatinization under high temperature and moisture, which increased the proportion of RDS [35]. Similar results have been reported by Gao et al., who observed higher GI values in steamed chestnuts compared with stir-fried or baked samples [10]. Parallel findings were also noted in taro, where boiling produced higher RDS than roasting, microwaving, or frying [36]. These observations suggest that cooking methods significantly modulate the glycemic response of chestnuts. Collectively, these findings demonstrate that cooking methods markedly influence the in vitro digestibility and glycemic properties of chestnuts.

### 3.3. Changes in Microstructure

**Figure 3:**
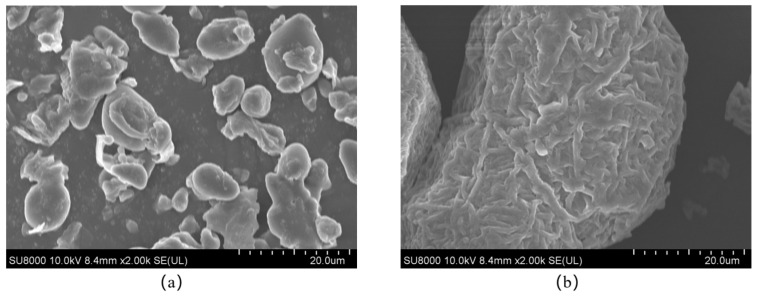

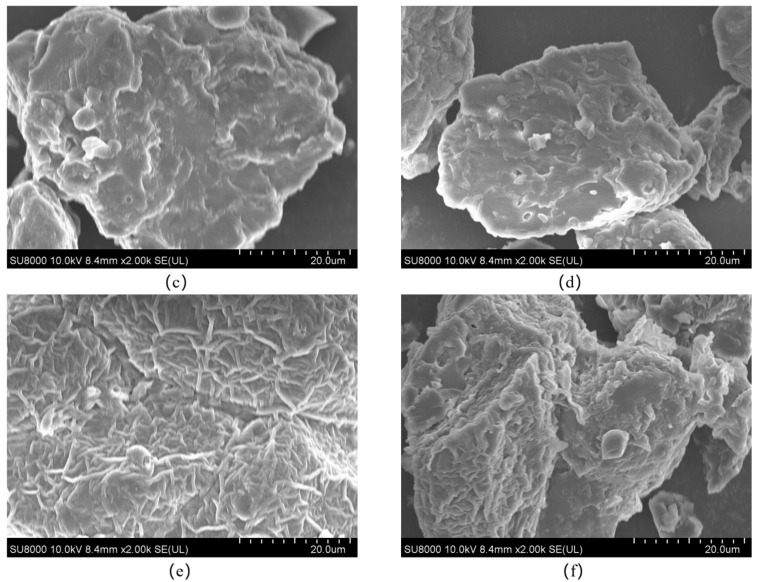
Changes in the microstructure of chestnuts as a function of cooking method. Images by SEM using a magnification 2000 times. (**a**) Raw chestnuts; (**b**) stir-fried chestnuts; (**c**) sugar-stir-fried chestnuts; (**d**) baked chestnuts; (**e**) steamed chestnuts; (**f**) boiled chestnuts.

As shown in Figure 3a–f, SEM analysis further clarified the relationship between starch morphology and digestibility in raw chestnuts and chestnuts treated with different cooking methods. Raw chestnuts (Figure 3a) exhibited predominantly round or oval granules with surface attachments, possibly proteins or cellulose, which is similar to the observation results in the previous studies of raw chestnut [37], raw oats [6], and raw triticale porridge [29]. After cooking, significant morphological changes were observed. Steamed (Figure 3e) and boiled chestnuts (Figure 3f) exhibited rough and fragmented structures, possibly due to water penetration into starch granules, causing expansion and cell wall rupture, which enhanced digestibility [38]. Stir-fried (Figure 3b), sugar-stir-fried (Figure 3c), and baked chestnuts (Figure 3d) also showed signs of gelatinization and rupture, in line with findings for Pleactranthus esculentus starch, which gelatinized within 55–80 °C during cooling [39]. These effects may be attributed to the high temperature reducing the moisture content, inducing starch chain cleavage [6]. In sugar-stir-fried chestnuts, the presence of reducing sugars further decreased water activity, accelerating the rupture of the starch chain and enhancing digestibility [40]. Although stir-fried (Figure 3b) and steamed (Figure 3e) chestnuts exhibited similar SEM morphology, with visible starch granule destruction, there are differences in their underlying mechanisms. In the process of stir-frying chestnuts, the high temperature and limited moisture causes a partial gel. In the process of steaming, high temperature and high humidity led to more thorough gel and particle expansion cracking [41]. Although they share some similarities in SEM, there are still significant differences in the texture, digestibility, and flavor of their final products.

In summary, moisture and temperature during cooking significantly affect chestnut starch gelatinization and Maillard reactions, thereby influencing texture, antioxidant activity, and glycemic response. High-temperature and high-humidity environments can promote the complete gelatinization of starch, destroy crystal structure, reduce chestnut hardness, and improve adhesion, while leading to increased digestibility and GI. High-temperature and low-humidity treatment can lead to partial gelatinization and water loss, forming a dense microstructure, increasing hardness, and reducing adhesion. The microstructure is closely related to the RS content, and the more severe the particle rupture, the more enzyme hydrolysis sites are exposed, and the lower the RS content. High temperatures also promote cell wall rupture and phenolic release, while intensifying the Maillard reaction. The resulting Maillard products not only enhance antioxidant activity [32], but may also accelerate starch hydrolysis, explaining the observed parallel trends between GI and antioxidant capacity [42].

### 3.4. Flavor by E-Nose Analysis

**Figure 4:**
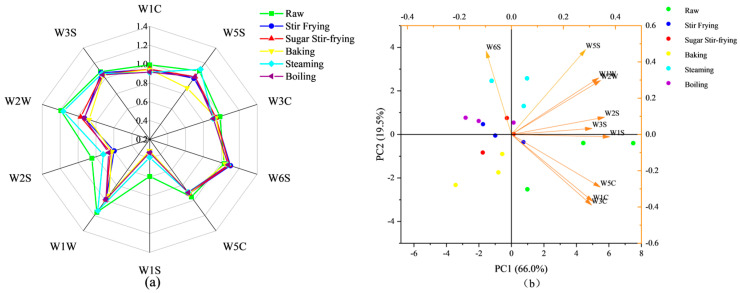
The radar chart (**a**) and PCA bioplot diagram (**b**) based on E-nose.

Figure 4 shows the flavor profiles of different chestnuts obtained using an electronic nose. The radar plot (Figure 4a) illustrates sensor responses across the raw chestnuts and chestnuts processed by different cooking methods. All samples showed a higher sensitivity in five sensors, including W1C (aromatic compounds, benzene), W3C (ammonia), W5C (short-chain alkanes), W3S (long-chain alkanes), and W6S (hydrides). Raw and steamed chestnuts exhibited comparable sensitivities to volatile compounds, with the most pronounced responses detected in W1W (aldehydes), W2W (alcohols), and W5S (nitrogen oxides). However, steamed chestnuts had lower sensitivities to W1S (methyl) and W2S (ketones) than raw chestnuts, possibly due to the degradation of volatile compounds during heating [43]. Stir-fried and sugar-stir-fried chestnuts exhibited nearly identical flavor responses, whereas baked chestnuts showed the lowest response value for W5S. These differences can be attributed to the specific processing techniques, which are consistent with previous research results on heat-treated nuts such as almonds, walnuts, cashews, and hazelnuts, where low-temperature treatments help preserve flavor substances, baking enhances sweetness, and high temperatures prompt flavor loss [26].

PCA further classified chestnut samples based on volatile profiles (Figure 4b). The cumulative contribution of the first two principal components reached 85.5%. PC1 exhibits the strongest correlation with W1S, accounting for 66.0%. PC2 shows the highest correlation with W6S, contributing to 19.5%, effectively capturing the majority of flavor variation. PCA separated chestnuts into five distinct groups: steamed chestnuts are mainly distributed in the first quadrant; boiled chestnuts are mainly distributed in the second quadrant; stir-fried chestnuts and sugar-stir-fried chestnuts are distributed near the origin, and their flavors are relatively similar; and baked chestnuts and raw chestnuts are gathered in the third and fourth quadrants, respectively. These results demonstrate that processing methods exert a significant influence on the flavor characteristics of chestnuts. Nonetheless, the electronic nose system detected only minor differences among stir-fried, sugar-stir-fried, and boiled chestnuts.

### 3.5. Volatile Compound Profile Analysis

#### 3.5.1. Overall Analysis of GC-IMS Results

**Figure 5:**
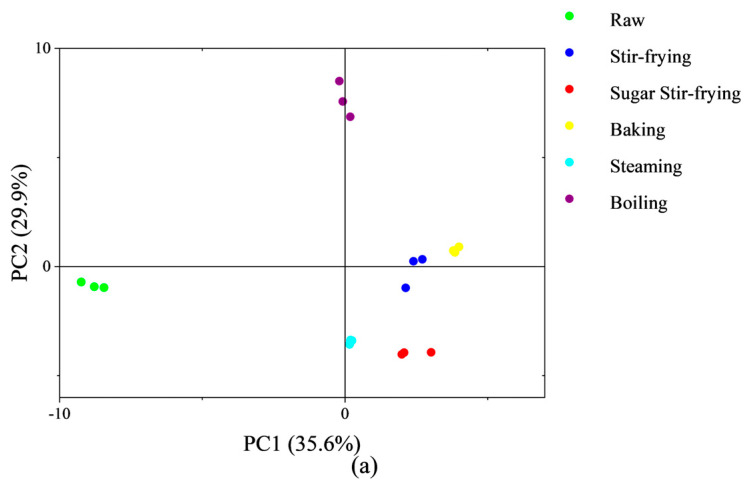

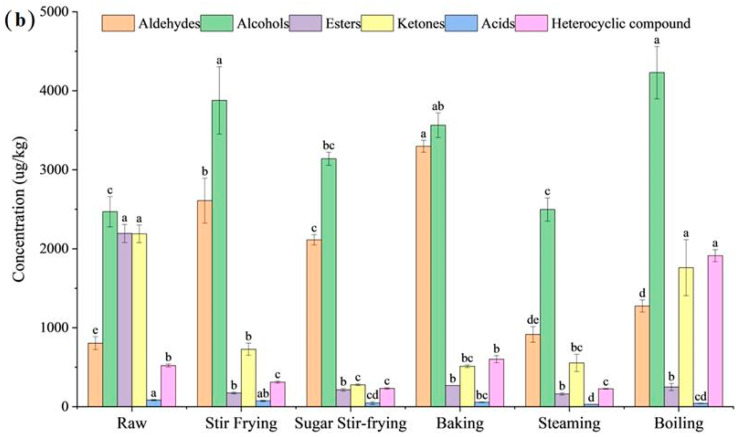
The concentration of PCA bioplot diagram (**a**), volatile compounds (**b**) of different chestnuts based on GC–IMS. Column in each class with different letters are significantly different (*p* < 0.05).

To further explore the impacts of different cooking methods on chestnut flavor, GC-IMS was employed to analyze the volatile compounds. Figure 5a shows the result of the PCA of GC-IMS data. It was found that the cumulative contribution of the first two principal components reached 65.5%. PC1 exhibits the strongest correlation with 3-Methyl-3-buten-1-ol, accounting for 35.6%; PC2 shows the highest correlation with nonanal, contributing to 29.9%, capturing the majority of flavor variation among samples. Raw chestnuts were located in the third quadrant, whereas steamed and boiled chestnuts, which retain relatively high moisture levels, occupied the lower and upper halves of the Y-axis, respectively. Their distribution possibly reflects the influence of starch gelatinization on volatile release. Stir-fried and sugar-stir-fried chestnuts, sharing similar cooking processes, clustered along the same vertical plane. Baked and stir-fried chestnuts also showed strong similarity, with their volatiles predominantly shaped by high temperature, oxygen exposure, and the nutrient composition of chestnuts [12].

A total of 34 volatile compounds were identified (Table S2), comprising eight aldehydes, eleven alcohols, four esters, five ketones, one acid, four heterocyclic compounds, and one other substance. Their relative concentrations are shown in Figure 5b. It was found that aldehydes were the relatively abundant group after cooking. Raw chestnuts contained 804.53 µg/kg, which increased significantly in all processed samples, similar to heat-treated coix seed [25], reaching the highest level in baked chestnuts (3297.48 µg/kg), followed by stir-fried (2608.89 µg/kg), sugar-stir-fried (2112.35 µg/kg), boiled (1276.04 µg/kg), and steamed chestnuts (916.37 µg/kg). This increase is primarily attributed to the Maillard reaction, which produces various aldehydes [44]. Aldehydes contribute fruity, fatty, and nutty notes [45], thereby enhancing the flavor of processed chestnuts.

Alcohols also played a key role in the chestnuts’ aroma, mainly originating from lipid oxidative degradation [46]. The total alcohol content in raw chestnuts (2468.72 µg/kg) increased significantly after cooking, with the highest levels found in boiled chestnuts (4230.03 µg/kg), followed by stir-fried (3878.08 µg/kg), baked (3564.22 µg/kg), sugar-stir-fried (3139.79 µg/kg), and steamed chestnuts (2497.98 µg/kg). This increase may be linked to the chestnuts’ high carbohydrate content, which provides precursors for amino acid synthesis. These amino acids are converted to α-ketoacids and subsequently transformed into alcohols through decarboxylation and reduction reactions [47]. Among the 16 detected alcohols, 1-octen-3-ol was also found in the fried chestnut [21], which imparted the characteristic aroma of chestnut mushrooms [48], generated via β-oxidation of linoleic acid [46], while other alcohols contributed fruity aromas.

Esters, responsible for fruity and floral notes [45], decreased significantly after cooking, similar to corn under different treatments [43]. Raw chestnuts contained 2193.32 µg/kg of esters, while steamed chestnuts retained the lowest content (163.92 µg/kg). Possibly, this decline is due to high-temperature oxidation and decomposition of esters, generating aldehydes and ketones, which explains the corresponding increase in aldehydes [43]. Cooking also caused a significant reduction in ketone content. In particular, sugar-stir-fried chestnuts retained only 12.73% of their initial ketone concentration, possibly due to heat-induced reduction reactions [43]. In contrast, boiled chestnuts exhibited a significantly higher concentration of heterocyclic compounds compared with other treatments (*p* < 0.05). Boiling, as a moist-heat process, prompts non-enzymatic oxidation, accelerating fatty acid degradation and generating volatile compounds with greater aromatic potency [43]. This pathway imparts roasted and earthy flavor attributes. Pyrazines, common aromatic compounds in roasted nuts, have also been detected in roasted almonds [49]. Notably, pyrazines, such as 2,6-dimethylpyrazine, which is a hallmark of the roasting aroma, have been identified in chestnuts as well. These compounds are formed through the reaction of α-dicarbonyl intermediates during the Maillard reaction [50]. Meanwhile, p-cresol, a distinctive aromatic compound, arises from the transformation of volatiles through Maillard and caramelization processes [1].

In summary, cooking markedly altered the volatile profile of chestnuts. Stir-frying, sugar stir-frying, and baking significantly increased aldehyde and alcohol contents, and boiling enhanced the abundance of volatiles. These changes appear to be driven by the combined effects of the Maillard reaction, lipid oxidation, and volatilization of aroma compounds at high temperatures [12].

#### 3.5.2. GC-IMS Fingerprint Analysis

**Figure 6:**
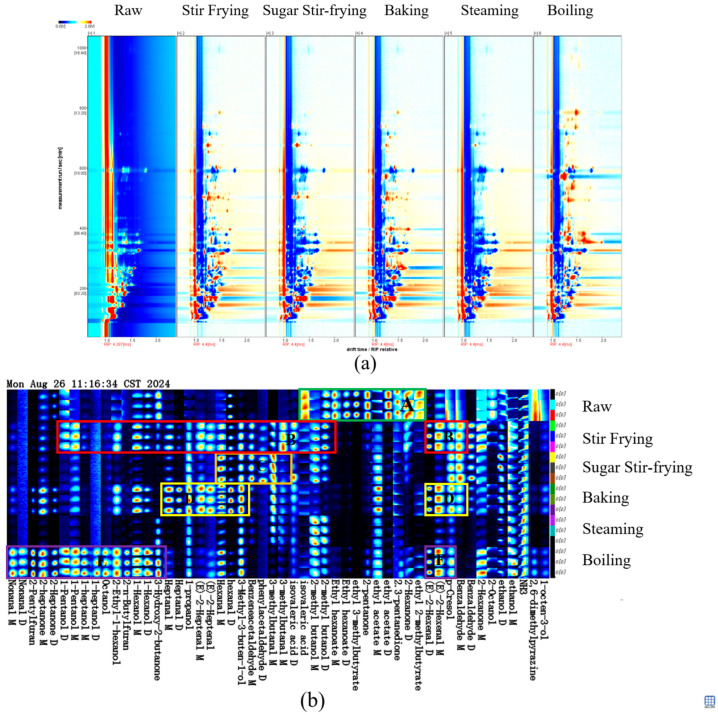
Two-dimensional graph (**a**), fingerprint (**b**) of different chestnuts based on GC–IMS.

Figure 6a presents the two-dimensional GC-IMS spectrum of chestnuts, where signal intensity (color depth) reflects compound concentration. Multiple dimeric substances were identified in chestnuts, including nonanal, phenylacetaldehyde, heptanol, ethyl hexanoate, and isovaleric acid, among others, depending on their chemical properties and abundance [20]. Meanwhile, to further elucidate the differences in volatile compounds across treatments, a fingerprint profile was constructed (Figure 6b). It was found that the compounds in region A were particularly abundant in raw chestnuts, comprising nine volatiles, including ethyl 2-methylbutyrate, 2-hexanone, 2,3-pentanedione, ethyl acetate, isovaleric acid, 2-methylbutanol, 2-pentanone, ethy 3-methylbutyrate, and ethyl caproate. These compounds contribute fresh and fruity aroma notes [50]. Notably, isovaleric acid was consistently detected across raw chestnuts and chestnuts processed by different cooking methods, with the highest concentration in raw samples.

The primary volatiles in stir-fried, sugar-stir-fried, and baked chestnuts were quite similar, including benzaldehyde, p-cresol, benzeneacetaldehyde, hexanal, and 2-hexenal, which are known contributors to fruity aromas [25]. Unique compounds such as 1-pentanol M and 1-pentanol D were detected in stir-fried chestnuts (region B), imparting balsamic notes. In stir-fried chestnuts, alcohols in region B increased significantly, likely due to ester hydrolysis [46]. In contrast, for sugar-stir-fried chestnuts (region C), low concentrations of 2-hexenalbenzaldehyde and benzeneacetaldehyde were observed, which arose from the Strecker degradation of phenylalanine [50]. Aldehydes and alcohols in region C decreased significantly after sugar stir-frying, possibly because sugar decomposition generated oxidizing intermediates that reacted with these compounds, reducing their abundance [51]. Baked chestnuts (region D) shared greater similarity with stir-fried chestnuts (region B), but differed significantly from sugar-stir-fried samples (region C). Steamed chestnuts were characterized by compounds such as 3-hydroxy-2-butanone, 3-methyl-3-buten-1-ol, isovaleraldehyde, and 2-methylbutanol, which contribute milky, sweet, fruity, floral, and fatty notes [25]. However, the diversity of volatiles in steamed chestnuts was relatively limited, likely because small-molecular compounds evaporated with water during high-temperature steaming [43].

The fingerprint profile of boiled chestnuts (region E) diverged distinctly from other treatments, encompassing heptanol, octanol, amyl alcohol, 2-heptanone, nonanaldehyde, 2-ethylhexanol, 2-butylfuran, and 2-pentylfuran. These volatiles impart fresh, fruity, fatty, and earthy aromas [25]. Notably, these two heterocyclic compounds were typically generated via the Maillard reaction and have also been found in heat-treated pumpkin seeds, while nonanaldehyde predominantly arose from fatty acid oxidation [50]. Collectively, isovaleric acid, 2-methylbutanol, 2-hexenal, and heterocyclic compounds, such as 2-butylfuran and 2-pentylfuran, appeared to be the key volatiles defining the distinctive flavor profiles of chestnuts processed by different cooking methods.

#### 3.5.3. VIP and ROAV Analysis Based on GC-IMS

**Figure 7:**
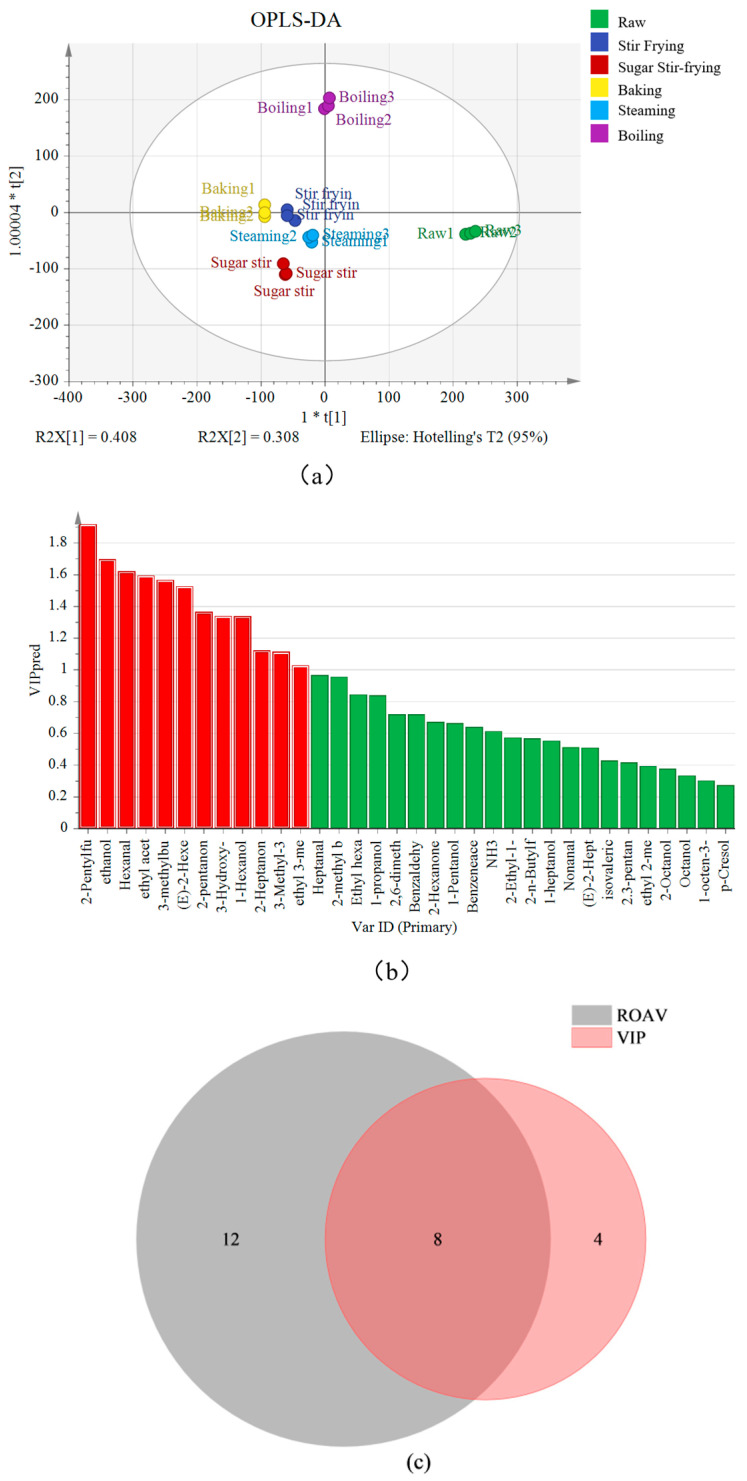
OPLS-DA (**a**), VIP (**b**), and VIP and ROAV Venn diagram (**c**) of different chestnuts based on GC–IMS. In (**b**), the red bars represent substances with VIP > 1; the green column represents substances with VIP < 1.

It establishes correlations between multivariate data and independent variables, generating variable importance in projection (VIP) values, where a higher VIP value indicates a greater contribution to the model [25]. The OPLS-DA discrimination models for various chestnut samples are shown in Figure 7a. The cumulative explained variance of the model was 71.6%: discriminant factor 1 exhibits the strongest correlation with ethyl acetate and 2-pentanone, accounting for 40.8%; discriminant factor 2 shows the highest correlation with 2-pentylfuran, contributing to 30.8%, indicating a strong fit. The R values of the first and second components were 40.8% and 30.8%, respectively. Overall, the classification results obtained from the OPLS-DA model were highly consistent with those of the PCA.

The key volatile compounds associated with different chestnut samples were identified using VIP values (Figure 7b). Compounds with VIP > 1 were considered to have a significant effect on the flavor profiles of chestnuts. In total, 12 compounds were found to be with VIP > 1, including 2-pentylfuran (1.92), ethanol (1.7), hexanal (1.62), ethyl acetate (1.53), (E)-2-hexenal (1.53), 3-methylbutanal (1.57), 2-pentanone (1.37), 3-hydrox -2-butanone (1.34), 1-hexanol (1.34), 2-heptanone (1.12), 3-methyl-3-buten-1-ol (1.11), and ethyl 3-methylbutyrate (1.03). These compounds serve as key markers for differentiating chestnuts processed by various cooking methods and possibly play an important role in shaping their characteristic flavor profiles.

The identification of key flavor substances based on ROAV > 1 and VIP > 1 has been applied to various foods, such as congou black tea [52] and dark tea [53]. As shown in Figure 7c, based on the combined criteria of VIP > 1 and ROAV > 1, eight key volatile compounds responsible for flavor differences among chestnut samples processed by different cooking methods were identified (Table S3). These compounds were mainly products of the Maillard reaction and lipid oxidation. Among the eight aldehyde compounds detected, three compounds were recognized as key contributors. Hexanal, mainly derived from the oxidative degradation of unsaturated fatty acids [45], was twice as abundant in baked as in raw chestnuts, imparting grassy, fatty, and fruity notes. (E)-2-hexenal, formed from the oxidative degradation of ethyl hexanoate [50], also played a significant role, particularly in stir-fried and baked chestnuts. In addition, 3-methylbutyraldehyde was also a key contributor to flavor. This compound is the product of the Strecker degradation pathway of the Maillard reaction [54]. As shown in Table S3, its content was significantly increased in stir-fried and sugar-stir-fried chestnuts. As reported, 3-methylbutyraldehyde is beneficial for enhancing caramel- and roasted-nut-like aromas, which has been confirmed in the study of roasted almonds [49].

Four ester compounds were identified, among which ethyl 3-methylbutyrate and ethyl acetate were most abundant in raw chestnuts, contributing fruity, sweet, and slightly sour notes [45]. The ROAVs and VIP values of these esters exceeded 1, confirming their dominant role in shaping the sensory characteristics of chestnuts.

Three ketones were also identified as key flavor substances. 2-heptanone, found at its highest concentrations in boiled chestnuts, imparted a fruity aroma [25]. In contrast, 2-pentanone was most abundant in raw samples, where it contributed a fresh fruity note [50], making for a defining flavor of raw chestnuts. 3-hydroxy-2-butanone, present in high concentrations in raw, steamed, and boiled chestnuts, imparted a buttery, fatty aroma. Moreover, the content of 2-pentylfuran increased significantly in baked and boiled chestnuts, with ROAVs and VIP values both exceeding 1, underscoring its importance in contributing green, fruity, and roasted notes [45]. Similarly, high ROAVs of 2-pentylfuran were also found in heat-treated corn [43].

In summary, cooking methods significantly affected the volatile profiles of chestnuts, with high temperatures, oxygen exposure, lipid oxidation, and the Maillard reaction driving the formation of complex aroma characteristics [12]. In particular, heterocyclic compounds from the Maillard reaction and alcohols from lipid oxidation play crucial roles in flavor development [46]. Volatile compounds, with ROAVs and VIP values both greater than 1, emerge as key determinants of the distinctive aromatic, fruity, and caramel-like profiles of chestnuts prepared by different cooking methods.

### 3.6. Sensory Evaluation

**Figure 8:**
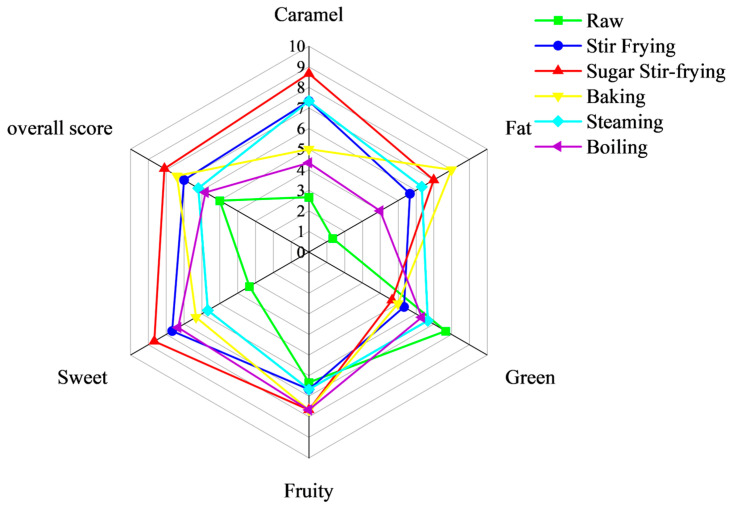
Radar plots of sensory scores of different chestnuts based on GC–IMS.

Figure 8 shows the radar chart of sensory evaluation, revealing the shaping effect of different cooking methods on the flavor characteristics of chestnuts. Among them, the green flavor of raw chestnuts is stronger, and their sweetness and caramel aroma ratings are lower. This might be because the only acidic compound detected, isovaleric acid, is most abundant in raw chestnuts (84.44 µg/kg). 2-pentanone has the highest content in raw chestnuts (968.63 µg/kg), and it has a fresh and fragrant smell. Electronic nose detection and sensory evaluation radar charts both show that the flavor characteristics of sugar-stir-fried chestnuts are similar to those of stir-fried chestnuts. However, the caramel aroma and sweetness intensity of sugar-stir-fried chestnuts are significantly higher than those of stir-fried chestnuts. This might be because the Maillard reaction of sugar-stir-fried chestnuts was stronger, with an increased content of 3-methylbutyraldehyde, which significantly enhanced the caramel aroma. At the same time, compared with traditional stir-frying methods, adding maltose during the sugar-fried chestnut process can further enhance the sweetness. The response value of the W5S sensor is the lowest for baked chestnuts, which can correspond to the results of lipid oxidation [55]. Moreover, baked chestnuts contain a high concentration of hexanal and (E)-2-hexenal, giving them a distinct fatty flavor [50]. In contrast, steamed and boiled chestnuts shared comparable sensory characteristics, both dominated by fruity and green notes. Compounds such as ethyl acetate, 2-heptanone, 2-pentanone, and 2-pentylfuran contributed substantially to these attributes [50].

In terms of overall preference, sugar-stir-fried chestnuts scored the highest, followed by baked, stir-fried, steamed, boiled, and raw chestnuts. This indicates that the caramel, nutty aroma, and moderate sweetness brought by the Maillard reaction may be more favored by the sensory evaluators. However, large-scale consumer acceptance research is still needed in the future to fully consider the impact of nutrition, flavor, antioxidant activity, GI response, and other factors on product acceptability.

## 4. Conclusions

This study systematically investigated the effects of different cooking methods on the physicochemical properties of chestnuts. The results showed that cooking methods significantly affected the chestnut quality (*p* < 0.05). Among them, the glycemic index (GI) of baked chestnuts was significantly lower than that of other cooked chestnuts. Steaming and boiling promoted sufficient water absorption and starch gelatinization, thereby forming a soft texture and bright color. In addition, the chestnuts after sugar stir-frying showed the highest antioxidant activity and a rich caramel flavor. Meanwhile, GC-IMS analysis identified eight key volatile compounds (including hexanal, (E)-2-hexenal, 3-methylbutanal, ethyl 3-methylbutyrate, ethyl acetate, 2-pentanone, 3-hydroxy-2-butanone, and 2-pentylfuran), clarifying the material basis of different cooking flavors. This study provided a new insight into how cooking methods affect the nutritional and flavor characteristics of chestnuts, thereby serving as a scientific basis for optimizing processing methods. Future research should include large-scale consumer acceptance tests to directly verify inferences based on instrument and sensory evaluations, and provide more direct evidence for product marketization.

## Figures and Tables

**Table 1:** Texture characteristics, moisture content, chromaticity, and digestive characteristics (RDS, SDS, RS, GI) of chestnuts cooked using different methods.

	Raw	Stir Frying	Sugar Stir-Frying	Baking	Steaming	Boiling
Pictures	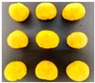	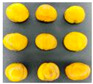	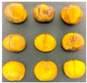	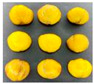	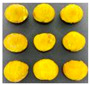	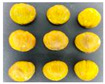
	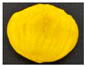	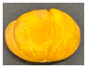	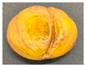	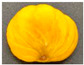	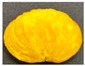	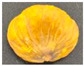
Hardness (N)	143.72 ± 5.67 ^a^	79.68 ± 2.08 ^c^	84.75 ± 9.15 ^b^	89.13 ± 3.58 ^b^	38.39 ± 2.45 ^d^	33.17 ± 0.73 ^d^
Adhesiveness (N.mm)	0.03 ± 0.00 ^ab^	0.03 ± 0.01 ^ab^	0.02 ± 0.01 ^b^	0.02 ± 0.01 ^b^	0.04 ± 0.02 ^a^	0.04 ± 0.00 ^a^
Cohesiveness (%)	0.63 ± 0.06 ^a^	0.43 ± 0.06 ^b^	0.40 ± 0.00 ^b^	0.37 ± 0.06 ^b^	0.27 ± 0.06 ^c^	0.28 ± 0.01 ^c^
Springiness (mm)	0.52 ± 0.05 ^b^	0.63 ± 0.05 ^ab^	0.68± 0.24 ^a^	0.69 ± 0.29 ^a^	0.58 ± 0.16 ^ab^	0.43 ± 0.15 ^c^
Gumminess (N)	59.27 ± 4.33 ^a^	27.88 ± 2.88 ^b^	25.46 ± 8.50 ^b^	24.20 ± 2.48 ^b^	23.57 ± 1.74 ^bc^	21.28 ± 0.35 ^c^
Chewiness (mJ)	48.93 ± 9.47 ^a^	17.63 ± 5.94 ^b^	12.97 ± 23.31 ^c^	20.93 ± 8.50 ^b^	7.70 ± 2.31 ^d^	5.20 ± 0.46 ^d^
Moisture content (%)	51.79 + 1.39 ^c^	43.24 ± 1.46 ^e^	47.18 ± 0.07 ^d^	34.75 ± 0.20 ^f^	53.45+ 1.07 ^b^	57.13 + 1.24 ^a^
L*	67.62 ± 4.16 ^a^	30.62 ± 0.21 ^d^	23.05 ± 0.14 ^e^	34.69 ± 0.82 ^c^	40.07 ±0.27 ^b^	38.52 ± 1.51 ^b^
a*	3.94 ± 0.79 ^b^	6.25 ± 0.25 ^a^	6.40 ± 0.06 ^a^	6.97 ± 0.25 ^a^	6.39 ± 0.07 ^a^	6.44 ± 0.26 ^a^
b*	19.20 ± 1.37 ^a^	8.59 ± 0.06 ^d^	8.08 ± 0.11 ^d^	12.93 ± 0.29 ^b^	13.52 ± 0.67 ^b^	10.95 ± 1.06 ^c^
∆E	-	38.81 ± 0.57 ^ab^	46.00 ± 4.42 ^a^	33.67 ± 4.48 ^c^	28.26 ± 4.75 ^c^	30.36 ± 4.32 ^c^
RDS%	5.81 ± 0.33 ^e^	19.58 ± 0.24 ^c^	20.52 ± 0.21 ^c^	17.48 ± 0.29 ^d^	29.95 ± 0.41 ^a^	26.32 ± 0.21 ^b^
SDS%	11.99 ± 0.19 ^d^	32.59 ± 0.11 ^bc^	36.17 ± 0.17 ^a^	30.99 ± 0.08 ^c^	32.47 ± 0.15 ^bc^	33.35 ± 0.10 ^b^
RS%	82.0 ± 0.36 ^a^	47.83 ± 0.31 ^c^	43.31 ± 0.25 ^c^	51.53 ± 0.39 ^b^	37.58 ± 0.48 ^e^	40.33 ± 0.37 ^d^
GI	52.49 ± 0.49 ^e^	78.09 ± 0.81 ^c^	80.34 ± 0.24 ^c^	74.78 ± 0.43 ^d^	85.42 ± 0.99 ^a^	83.26 ± 0.31 ^b^

Note: L*—luminance; a*—red–green degree; b*—yellow–blue degree; ∆E—total color difference; RDS—rapidly digestible starch; SDS—slowly digestible starch; RS—resistant starch; GI—glycemic index. Values in each line with different letters are significantly different (*p* < 0.05).

## Data Availability

The original contributions presented in the study are included in the article; further inquiries can be directed to the corresponding author.

## Supplementary Materials

The following supporting information can be downloaded at: https://www.mdpi.com/article/10.3390/foods14244331/s1; Table S1: Sensor type and performance description of E-nose system; Table S2: Information on volatile compounds of different chestnuts identified by GC-IMS; Table S3: Information on key volatile compounds of different chestnuts identified by GC-IMS.

## Abbreviations

The following abbreviations are used in this manuscript:

GC-IMS	Gas chromatography–ion mobility spectrometry
RDS	Rapidly digestible starch
SDS	Slowly digestible starch
RS	Resistant starch
GI	Glycemic index
TPC	Total polyphenol content
DPPH	2,2-diphenyl-1-picrylhydrazyl
ABTS	2,2′-azino-bis(3-ethylbenzothiazoline-6-sulfonic acid
FRAP	Ferric Reducing Antioxidant Power
SEM	Scanning electron microscopy
ROAV	Relative odor activity value
PCA	Principal component analysis
OPLS-DA	Orthogonal partial least squares discriminant analysis
VIP	Variable importance in projection
