# Supplementary material for: Impact of Cooking Method on the Physicochemical Properties, Digestibility, and Sensory and Flavor Profiles of Chinese Chestnuts
Source: Foods. 2025 Dec 16;14(24):4331. doi: 10.3390/foods14244331 (PMC12732960; doi:10.3390/foods14244331)
Supplement: Supplementary file 1 [file foods-14-04331-s001.zip › foods-3979719-supplementary.docx]

**Table S1.** Sensor type and performance description of E-nose system.

| **Array number** | **Sensor name** | **Representative substance types** | **Performance description** |
| --- | --- | --- | --- |
| 1 | W1C | Aromatic | Aromatic components - Benzenes |
| 2 | W5S | Broad range | High sensitivity, very sensitive to nitrogen oxides |
| 3 | W3C | Aromatic | Sensitive aromatic components, ammonia |
| 4 | W6S | Hydrogen | Mainly selective towards hydrides |
| 5 | W5C | Arom-aliph | Aromatic components of short chain alkanes |
| 6 | W1S | Broad-methane | Sensitive to methyl groups |
| 7 | W1W | Sulphur-organic | Sensitive to sulfides |
| 8 | W2S | Broad-chlor | Sensitive to alcohols, aldehydes, and ketones |
| 9 | W2W | Sulph-chlor | Aromatic components, sensitive to organic sulfides |
| 10 | W3S | Methane-aliph | Sensitive to long-chain alkanes |

**Table S2. Information on volatile compounds of different chestnuts identified by GC-IMS.**

| NO. | Compound | CAS | Threshold | RI | Rt(sec) | Dt | Aroma description | Relative content (ug/kg) | | | | | | ROAV | | | | | | VIP |
| --- | --- | --- | --- | --- | --- | --- | --- | --- | --- | --- | --- | --- | --- | --- | --- | --- | --- | --- | --- | --- |
|  |  |  |  |  |  |  |  | Raw | Stir-frying | Sugar-stir-frying | Baking | Steaming | Boiling | Raw | Stir-frying | Sugar-stir-frying | Baking | Steaming | Boiling |  |
|  | Aldehyde |  |  |  |  |  |  |  |  |  |  |  |  |  |  |  |  |  |  |  |
| 1 | Nonanal | 124-19-6 | 0.045 | 1104.2 | 785.479 | 1.4844 | rose, citrus, strong, oily | 18.43±0.8 | 43.72±9.12 | 24.01±2.76 | 49.34±5.73 | 21.86±2.43 | 126.83±7.85 | 3.96 | 4.96 | 6.40 | 1.40 | 7.85 | 0.98 | 0.51 |
| 2 | Benzeneacetaldehyde | 122-78-1 | 0.04 | 1053.8 | 678.84 | 1.25966 | hyacinth, sweet fruity, almond, cherry, clover honey, cocoa | 17.88±8.16 | 112.92±7.15 | 140.6±14.42 | 50.48±8.31 | 41.93±1.97 | 14.34±1.48 | 4.32 | 14.41 | 42.16 | 1.62 | 16.94 | 0.14 | 0.64 |
| 3 | Benzaldehyde | 100-52-7 | 2 | 961.8 | 503.599 | 1.46806 | bitter almond, cherry, nutty | 48.12±8.93 | 143.17±3.7 | 147.78±18.88 | 83.37±2.81 | 15.83±1.72 | 11.65±0.26 | 0.23 | 0.37 | 0.89 | 0.05 | 0.13 | 0.00 | 0.72 |
| 4 | Heptanal | 111-71-7 | 0.02 | 898.9 | 396.13 | 1.34623 | fresh, aldehyde, fatty, green herbs, wine, fruity | 42.17±2.51 | 169.99±34.03 | 66.85±6.22 | 359.33±19.24 | 23.65±2.5 | 5.84±0.71 | 20.37 | 43.37 | 40.09 | 23.02 | 19.10 | 1.26 | 0.97 |
| 5 | Hexanal | 66-25-1 | 0.3 | 789.7 | 270.266 | 1.56424 | fresh, green, fat, fruity | 476.02±56.32 | 625.53±79.03 | 476.01±25.2 | 1179.79±27.31 | 120.61±10.11 | 9.55±0.8 | 15.33 | 10.64 | 19.03 | 5.04 | 6.50 | 0.37 | 1.62 |
| 6 | (E)-2-Hexenal | 6728-26-3 | 1 | 837.5 | 319.175 | 1.18665 | green, banana, fat | 144.07±14.66 | 535.65±101.35 | 48.43±3.54 | 541.86±10.38 | 58.93±4.53 | 4.73±0.1 | 1.39 | 2.73 | 0.58 | 0.69 | 0.95 | 0.16 | 1.53 |
| 7 | (E)-2-Heptenal | 18829-55-5 | 0.63 | 957.7 | 495.857 | 1.25867 | spicy, green vegetables, fresh, fatty | 9.66±1.41 | 69.68±6.19 | 27.64±4.14 | 77.56±2.69 | 13.7±0.93 | 67.75±2.9 | 0.15 | 0.56 | 0.53 | 0.16 | 0.35 | 0.01 | 0.51 |
| 8 | 3-methylbutanal | 590-86-3 | 0.5 | 651.2 | 167.446 | 1.40787 | Caramel, roasted, nut | 48.2±0.9 | 908.23±93.37 | 1181.04±71.62 | 955.76±42.82 | 619.85±104.63 | 12.99±0.12 | 0.93 | 9.27 | 28.33 | 2.45 | 20.03 | 0.09 | 1.57 |
|  | Alcohol |  |  |  |  |  |  |  |  |  |  |  |  |  |  |  |  |  |  |  |
| 9 | Octanol | 111-87-5 | 0.5 | 1081.3 | 734.995 | 1.47607 | citrus, sweet, herbs, waxy, rose, mushroom | 10.62±0.67 | 17.76±2.46 | 14.17±1.57 | 15.36±1.44 | 12.02±0.84 | 58.95±4.1 | 0.21 | 0.18 | 0.34 | 0.04 | 0.39 | 0.04 | 0.34 |
| 10 | 1-heptanol | 111-70-6 | 2.4 | 978.3 | 536.517 | 1.77584 | grape, fruity, wine, violet, peony | 29.88±2.13 | 52.56±8.96 | 20.43±2.04 | 25.28±2.69 | 20.8±2.02 | 153.54±4.43 | 0.12 | 0.11 | 0.10 | 0.01 | 0.14 | 0.02 | 0.56 |
| 11 | 1-Pentanol | 71-41-0 | 4.5 | 760.6 | 242.956 | 1.25449 | balsamic | 30.76±2.25 | 108.57±12.65 | 38.81±1.4 | 48.22±2.82 | 28.18±1.52 | 118.09±13.87 | 0.07 | 0.12 | 0.10 | 0.01 | 0.10 | 0.01 | 0.66 |
| 12 | 1-Hexanol | 111-27-3 | 8 | 866.8 | 353.53 | 1.64118 | fresh, fruity, wine, sweet, green | 359.2±30.66 | 452.47±67.83 | 76.46±2.66 | 455.74±27.92 | 65.37±7.98 | 897.15±21.79 | 0.43 | 0.29 | 0.11 | 0.07 | 0.13 | 0.03 | 1.34 |
| 13 | 2-methyl butanol | 137-32-6 | 6.25 | 724.8 | 212.744 | 1.47736 | roast onion, fruity, floral, wine | 210.22±5.03 | 287.56±19.03 | 208.21±11.67 | 79.47±2.07 | 376.71±15.21 | 244.57±29.25 | 0.32 | 0.23 | 0.40 | 0.02 | 0.97 | 0.01 | 0.96 |
| 14 | 1-propanol | 71-23-8 | 5.5 | 592.6 | 142.02 | 1.24314 | alcohol, pungent | 115±4.93 | 263.92±34.11 | 278.25±13.31 | 429.33±6.89 | 238.24±34.51 | 255.62±36.44 | 0.20 | 0.24 | 0.61 | 0.10 | 0.70 | 0.01 | 0.84 |
| 15 | ethanol | 64-17-5 | 20 | 491 | 106.764 | 1.11766 | aromaticity | 1507.18±186.06 | 2001.4±268.53 | 1987.41±250.17 | 1604.8±178.37 | 1314.43±118.49 | 1750.42±257.82 | 0.73 | 0.51 | 1.19 | 0.10 | 1.06 | 0.03 | 1.7 |
| 16 | 3-Methyl-3-buten-1-ol | 763-32-6 |  | 749.9 | 233.431 | 1.41803 | sweet, fruity | 52±7.5 | 554.38±50.57 | 480.3±31.32 | 765.24±4.45 | 418.66±57.96 | 575.14±96.17 | / | / | / | / | / | / | 1.11 |
| 17 | 2-Ethyl-1-hexanol | 104-76-7 |  | 1010.7 | 599.223 | 1.41727 | citrus, fresh floral, greasy | 37.94±5.25 | 88.76±20.86 | 15.86±1.69 | 84.49±9.63 | 10.47±0.17 | 116.92±3.36 | / | / | / | / | / | / | 0.57 |
| 18 | 2-Octanol | 123-96-6 |  | 1007.1 | 593.027 | 1.45147 | fresh, herbaceous, earthy | 70.9±10.02 | 29.74±4.7 | 7.75±0.94 | 22.79±2.33 | 7.07±0.94 | 31.18±2.01 | / | / | / | / | / | / | 0.38 |
| 19 | 1-octen-3-ol | 3391-86-4 | 0.05 | 984.5 | 549.312 | 1.1563 | mushroom, lavender, rose, hay | 45.02±10.2 | 20.99±2.91 | 12.14±1.47 | 33.51±1.3 | 6.04±0.79 | 28.45±2.21 | 8.70 | 2.14 | 2.91 | 0.86 | 1.95 | 0.18 | 0.3 |
|  | Ester |  |  |  |  |  |  |  |  |  |  |  |  |  |  |  |  |  |  |  |
| 20 | Ethyl hexanoate | 123-66-0 | 0.075 | 1008.6 | 595.576 | 1.34623 | pineapple, fruity, wine | 360.34±41.04 | 37.82±6.67 | 18.76±2.65 | 29.76±2.67 | 20.64±1.08 | 46.13±2.09 | 46.41 | 2.57 | 3.00 | 0.51 | 4.45 | 0.19 | 0.85 |
| 21 | ethyl 3-methylbutyrate | 7452-79-1 | 0.2 | 845.8 | 328.543 | 1.64437 | apple, banana, sour, and sweet | 506.11±50.42 | 24.88±6.29 | 12.04±0.82 | 25.79±1.06 | 12.21±1.07 | 21.95±4.06 | 24.44 | 0.63 | 0.72 | 0.17 | 0.99 | 0.03 | 1.03 |
| 22 | ethyl 2-methylbutyrate | 7452-79-1 | 0.2 | 845.4 | 328.117 | 1.25927 | apple | 80.63±7.04 | 12.35±2.52 | 3.15±0.6 | 14.78±0.72 | 3.59±0.67 | 18.24±2.78 | 3.89 | 0.32 | 0.19 | 0.09 | 0.29 | 0.03 | 0.4 |
| 23 | ethyl acetate | 141-78-6 | 11 | 605.8 | 147.396 | 1.33386 | fresh, fruity, sweet, grassy | 1246.24±49.34 | 99.39±9.6 | 180.47±16.41 | 199.39±2.4 | 127.48±14.41 | 163.67±38.03 | 1.09 | 0.05 | 0.20 | 0.02 | 0.19 | 0.00 | 1.6 |
|  | Ketone |  |  |  |  |  |  |  |  |  |  |  |  |  |  |  |  |  |  |  |
| 24 | 2-heptanone | 110-43-0 | 0.6 | 888.8 | 381.607 | 1.62355 | pear, banana, fruity, slight medicinal fragrance | 190.58±21.59 | 90.53±11.77 | 66.8±3.66 | 271.46±14.48 | 35.42±2.83 | 548±52.29 | 3.07 | 0.77 | 1.34 | 0.58 | 0.95 | 0.28 | 1.12 |
| 25 | 2.3-pentanedione | 600-14-6 | 0.005 | 711.9 | 202.741 | 1.21345 | sweet, cream, caramel, nuts, cheese | 87.19±7.86 | 11.97±0.85 | 6.74±0.65 | 15.98±0.63 | 5.67±0.22 | 44.85±4.81 | 168.45 | 12.22 | 16.17 | 4.09 | 18.32 | 2.80 | 0.42 |
| 26 | 2-pentanone | 107-87-9 | 7 | 675.2 | 179.135 | 1.36521 | acetone, fresh, sweet fruity, wine | 968.63±44.64 | 184.89±29.62 | 95.59±9.77 | 103.66±2.91 | 43.73±11.29 | 225.44±52.03 | 1.34 | 0.13 | 0.16 | 0.02 | 0.10 | 0.01 | 1.37 |
| 27 | 3-Hydroxy-2-butanone | 513-86-0 | 6 | 702.5 | 195.838 | 1.32975 | butter, cream，fat, | 810.58±63.76 | 337.4±28.98 | 61.33±6.87 | 96.3±3.03 | 454.81±97.72 | 868.75±245.94 | 1.31 | 0.29 | 0.12 | 0.02 | 1.22 | 0.05 | 1.34 |
| 28 | 2-Hexanone | 591-78-6 | 0.93 | 772.8 | 254.188 | 1.47811 | fruity, fungal, meaty, buttery | 131.86±10.81 | 103.49±15.81 | 48.21±2.2 | 25.56±1.09 | 15.52±1.16 | 73.66±12.86 | 1.37 | 0.57 | 0.62 | 0.04 | 0.27 | 0.02 | 0.67 |
|  | Acid |  |  |  |  |  |  |  |  |  |  |  |  |  |  |  |  |  |  |  |
| 29 | isovaleric acid M | 503-74-2 | 0.55 | 804.6 | 284.685 | 1.47045 | sour, foot sweat, cheese | 84.44±6.33 | 74.5±9.58 | 46.15±19.93 | 57.1±5.03 | 30.38±3.33 | 41.06±3.46 | 1.48 | 0.69 | 1.01 | 0.13 | 0.89 | 0.02 | 0.43 |
|  | Heterocycles |  |  |  |  |  |  |  |  |  |  |  |  |  |  |  |  |  |  |  |
| 30 | 2-Pentylfuran | 3777-69-3 | 0.0048 | 994.8 | 571.371 | 1.25004 | bean, fruity, earthy, green, roasted | 49.64±3.05 | 94.06±11.54 | 40.02±0.95 | 374.63±41.43 | 29.71±4.02 | 1539.73±49.25 | 100.00 | 100.00 | 100.00 | 100.00 | 100.00 | 100.00 | 1.92 |
| 31 | 2-n-Butylfuran | 4466-24-4 |  | 889 | 381.874 | 1.1795 | mild fruity, alcoholic, sweet, and spicy | 46.16±8.4 | 18.93±0.58 | 27.38±1.55 | 33.22±2.35 | 21.06±2.12 | 137.94±5.36 | / | / | / | / | / | / | 0.57 |
| 32 | 2,6-dimethylpyrazine | 108-50-9 | 0.1 | 920.8 | 430.663 | 1.13402 | roast, coffee, peanut, potato | 393.77±12.02 | 160.61±1.65 | 124.68±7.41 | 152.81±2.99 | 159.9±5.01 | 201.87±21.89 | 38.04 | 8.20 | 14.95 | 1.96 | 25.83 | 0.63 | 0.72 |
| 33 | p-Cresol | 106-44-5 | 0.01 | 1083.2 | 739.055 | 1.16012 | aromatic, cellar mud | 32.35±7.36 | 38.06±4 | 39.86±2.86 | 41.89±2.23 | 17.92±1.7 | 33.47±4.03 | 31.25 | 19.42 | 47.81 | 5.37 | 28.95 | 1.04 | 0.28 |
|  | Unidentified |  |  |  |  |  |  |  |  |  |  |  |  |  |  |  |  |  |  |  |
| 34 | NH3 | unidentified |  | 937.8 | 459.559 | 1.17346 |  | 2.40±0.08 | 4.4±0.21 | 5.14±0.07 | 4.14±0.24 | 8.37±0.66 | 3.58±0.37 | / | / | / | / | / | / | 0.62 |

Values were means ± standard deviation. /: not detecte.

**Table S3.** Information on key volatile compounds of different chestnuts identified by GC-IMS.

| NO. | Compound | CAS | Threshold | RI | Rt(sec) | Dt | Aroma description | Relative content (μg/kg) | | | | | | ROAV | | | | | | VIP |
| --- | --- | --- | --- | --- | --- | --- | --- | --- | --- | --- | --- | --- | --- | --- | --- | --- | --- | --- | --- | --- |
|  |  |  |  |  |  |  |  | Raw | Stir frying | Sugar stir frying | Baking | Steaming | Boiling | Raw | Stir frying | Sugar stir frying | Baking | Steaming | Boiling |  |
| 1 | Hexanal | 66-25-1 | 0.3 | 789.7 | 270.266 | 1.56424 | fresh, green, fat, fruity | 476.02±56.32 | 625.53±79.03 | 476.01±25.2 | 1179.79±27.31 | 120.61±10.11 | 9.55±0.8 | 15.33 | 10.64 | 19.03 | 5.04 | 6.50 | 0.37 | 1.62 |
| 2 | (E)-2-Hexenal | 6728-26-3 | 1 | 837.5 | 319.175 | 1.18665 | green, banana, fat | 144.07±14.66 | 535.65±101.35 | 48.43±3.54 | 541.86±10.38 | 58.93±4.53 | 4.73±0.1 | 1.39 | 2.73 | 0.58 | 0.69 | 0.95 | 0.16 | 1.53 |
| 3 | 3-methylbutanal | 590-86-3 | 0.5 | 651.2 | 167.446 | 1.40787 | Caramel, roasted, nut | 48.2±0.9 | 908.23±93.37 | 1181.04±71.62 | 955.76±42.82 | 619.85±104.63 | 12.99±0.12 | 0.93 | 9.27 | 28.33 | 2.45 | 20.03 | 0.09 | 1.57 |
| 4 | ethyl 3-methylbutyrate | 7452-79-1 | 0.2 | 845.8 | 328.543 | 1.64437 | apple, banana, sour, and sweet | 506.11±50.42 | 24.88±6.29 | 12.04±0.82 | 25.79±1.06 | 12.21±1.07 | 21.95±4.06 | 24.44 | 0.63 | 0.72 | 0.17 | 0.99 | 0.03 | 1.03 |
| 5 | ethyl acetate | 141-78-6 | 11 | 605.8 | 147.396 | 1.33386 | fresh, fruity, sweet, grassy | 1246.24±49.34 | 99.39±9.6 | 180.47±16.41 | 199.39±2.4 | 127.48±14.41 | 163.67±38.03 | 1.09 | 0.05 | 0.20 | 0.02 | 0.19 | 0.00 | 1.6 |
| 6 | 2-pentanone | 107-87-9 | 7 | 675.2 | 179.135 | 1.36521 | acetone, fresh, sweet fruity, wine | 968.63±44.64 | 184.89±29.62 | 95.59±9.77 | 103.66±2.91 | 43.73±11.29 | 225.44±52.03 | 1.34 | 0.13 | 0.16 | 0.02 | 0.10 | 0.01 | 1.37 |
| 7 | 3-Hydroxy-2-butanone | 513-86-0 | 6 | 702.5 | 195.838 | 1.32975 | butter, cream，fat, | 810.58±63.76 | 337.4±28.98 | 61.33±6.87 | 96.3±3.03 | 454.81±97.72 | 868.75±245.94 | 1.31 | 0.29 | 0.12 | 0.02 | 1.22 | 0.05 | 1.34 |
| 8 | 2-Pentylfuran | 3777-69-3 | 0.0048 | 994.8 | 571.371 | 1.25004 | bean, fruity, earthy, green, roasted | 49.64±3.05 | 94.06±11.54 | 40.02±0.95 | 374.63±41.43 | 29.71±4.02 | 1539.73±49.25 | 100.00 | 100.00 | 100.00 | 100.00 | 100.00 | 100.00 | 1.92 |

Values were means ± standard deviation. /: not detect.
